# HPV prevention awareness, vaccination willingness, and vaccine-related perceptions among middle-school girls in three Arab countries: an exploratory online survey

**DOI:** 10.3389/fpubh.2026.1902409

**Published:** 2026-08-03

**Authors:** Fatma Magdi Ibrahim, Ateya Megahed Ibrahim, Eman Abdelaziz Rashad Dabou, Soad Abd Elhameed, Victoria Hanson

**Affiliations:** 1 RAK College of Nursing, RAK Medical and Health Sciences University, Ras Al Khaimah, United Arab Emirates; 2 Faculty of Nursing, New Mansoura University, Mansoura, Egypt; 3 Nursing College, Prince Sattam Bin Abdulaziz University, Al-Kharj, Saudi Arabia; 4 Faculty of Nursing, Port Said University, Port Said, Egypt; 5 Faculty of Nursing, Alexandria University, Alexandria, Egypt; 6 Faculty of Nursing, Mansoura University, Mansoura, Egypt

**Keywords:** adolescents, Arab countries, cervical cancer prevention, exploratory survey, human papillomavirus, school health, vaccination willingness, vaccine confidence

## Abstract

**Background:**

Cervical cancer is largely preventable through human papillomavirus (HPV) vaccination, screening, and timely treatment; however, adolescent HPV literacy remains inconsistent. Egypt, Saudi Arabia, and the United Arab Emirates have different publicly documented HPV-vaccination and cervical-screening contexts. This study explored HPV and cervical cancer prevention awareness, selected Health Belief Model-informed beliefs, vaccination willingness, and vaccine-related perceptions among middle-school girls in these countries.

**Methods:**

An exploratory online cross-sectional survey was conducted among 300 students, with equal quota-like groups of 100 per country recruited through non-probability snowball sampling. Analyses were primarily descriptive; one complete-case exploratory logistic model examined factors associated with willingness to accept free HPV vaccination.

**Results:**

Overall, 89/300 participants (29.7%) had heard of HPV, 103/300 (34.3%) had heard of cervical cancer, and 49/300 (16.3%) were aware of cervical screening. School nurses were the most frequently reported HPV information source among source-item respondents (47/90; 52.2%). HPV-vaccine awareness was reported by 131/298 participants (44.0%), previous vaccination by 106/300 (35.3%), and willingness to accept free vaccination by 197/300 (65.7%). Responses to five negatively worded vaccine-perception items were predominantly neutral or reflected disagreement rather than consistent endorsement of distrust. The exploratory model generated several inverse associations with willingness, but these estimates are sample-specific and hypothesis-generating.

**Conclusion:**

Prevention awareness was limited despite relatively high stated willingness to accept free vaccination. The findings support culturally adapted, school-linked education and representative studies using validated measures, parent-linked information, and verified vaccination records.

## Introduction

Cervical cancer remains a major public health concern despite being largely preventable. The World Health Organization (WHO) estimated 660,000 new cervical cancer cases and 350,000 deaths in 2022, with the burden concentrated in settings where vaccination, screening, and treatment services are less accessible (1, 2). Persistent infection with high-risk HPV causes almost all cervical cancers, and HPV vaccination before exposure to HPV is a core component of prevention (2, 3).

Early adolescence is therefore a critical window for health education and vaccine communication. At this age, students and parents may be forming beliefs about cancer prevention, vaccine safety, and trust in health systems before routine vaccination opportunities are missed. School-based health education is particularly relevant because it can deliver age-appropriate information, engage parents, and connect adolescents with trusted health professionals (4).

Evidence from Arab and Muslim-majority settings has documented knowledge gaps, stigma linked to sexually transmitted infections, parental gatekeeping, and concerns about vaccine safety and effectiveness (5–7). However, most studies in the region have involved university students, adult women, or parents rather than younger adolescents (5–8). This leaves an important evidence gap for school-health planning.

Egypt, Saudi Arabia, and the United Arab Emirates were included because the investigators had academic and school-health networks in these countries and because their publicly documented HPV-prevention contexts differ (Table 1). These differences concern vaccination recommendations and program integration as well as cervical-screening guidance (9–11). The policy information is presented only to define the interpretive context; it was not measured as an individual exposure, may not reflect implementation or coverage, and cannot explain differences observed in this non-probability sample.

**Table 1:** Publicly documented HPV-vaccination and cervical-screening context in the three study countries.

Country	Publicly documented HPV-vaccination context	Publicly documented cervical-screening context
Egypt	HPV vaccine availability is documented, but regional policy tracking does not describe it as fully embedded in the routine national immunization program (9).	Publicly accessible national screening-policy documentation is less developed than in the other two settings; this study did not measure access or program exposure (9).
Saudi Arabia	Ministry of Health guidance recommends HPV vaccination for adolescents and describes vaccination as a principal prevention measure (10).	Ministry guidance recommends routine cervical screening; public recommendations should not be interpreted as verified population coverage (10).
United Arab Emirates	The national prevention plan targets HPV vaccination of 90% of girls before age 15 by 2030 (11).	The same plan specifies early cervical screening beginning at age 25 and is supported by national screening guidance (11).

The table summarizes publicly available policy information used for interpretation at the time of manuscript revision. It does not measure program implementation, access, uptake, or participants’ exposure during data collection.

The Health Belief Model (HBM) offers a useful lens for understanding preventive behavior through perceived susceptibility, severity, benefits, barriers, and cues to action (12–14). In this study, however, the available items support only descriptive analysis of selected HBM-informed beliefs and cues rather than a validated HBM scale. The objective was therefore to describe HPV and cervical cancer prevention awareness, self-reported HPV-vaccine awareness and uptake, selected HBM-informed beliefs, willingness to accept free vaccination, and vaccine-related perceptions in an exploratory online sample of middle-school girls in three Arab countries.

## Materials and methods

### Design and reporting

This was an exploratory cross-sectional online survey. The manuscript was prepared with reference to the Strengthening the Reporting of Observational Studies in Epidemiology (STROBE) guidance for cross-sectional studies (15). Because recruitment used online snowball methods, all estimates should be interpreted as sample-specific rather than population-representative.

### Setting and participants

The study included female middle-school students residing in Egypt, Saudi Arabia, or the United Arab Emirates. Eligibility criteria were self-identifying as female, current middle-school enrolment, residence in one of the three study countries, ability to understand the survey language, and electronic parent/guardian consent with student assent before participation. Age, school level, sex, and country of residence were self-reported and were not independently verified through school or health records.

### Sampling and recruitment

Participants were recruited through non-probability snowball sampling. An anonymous survey link was circulated through social media and WhatsApp networks and could be shared with eligible peers. Recruitment achieved 300 responses, with equal quota-like groups of 100 respondents per country. The target of 100 participants per country was feasibility-based and intended to create balanced groups for preliminary description; no probability-based sample-size calculation was performed. The study was not designed to provide national estimates or definitive cross-country comparisons.

### Ethical considerations for minors

Ethical approval was obtained from the Ethical Committee of Faculty of Nursing, Mansoura University (Ref. No. p.0370), the Scientific Research Ethics Committee, Prince Sattam Bin Abdulaziz University, Al-Kharj, Saudi Arabia (approval no. SCRB-267/2024), and the Research and Ethics Committee, RAK Medical and Health Sciences University, Ras Al Khaimah, United Arab Emirates (approval no. RAKMHSU REC 2023/24-F-N). The online information page described the study purpose, voluntary nature of participation, confidentiality protections, and the right to stop or skip questions. Electronic parent/guardian consent and student assent were requested before the questionnaire opened. No names, phone numbers, school identifiers, or vaccination records were collected. Because consent and eligibility were handled remotely, verification of parent/guardian identity and vaccination status was not possible; this is treated as a limitation.

### Questionnaire and measures

The questionnaire was developed for this study from literature on HPV, cervical cancer prevention, vaccination, and HBM constructs (4–8, 12–14). It included sociodemographic characteristics; awareness of HPV, cervical cancer, screening, and HPV vaccination; self-reported vaccine uptake and series completion; willingness to accept free vaccination; refusal reasons; selected HBM-informed beliefs and cues; and vaccine-perception items. Content validity, clarity, and cultural appropriateness were reviewed by experts in nursing, oncology, and public health, and the questionnaire was piloted with students who were not included in the final analysis.

Awareness and belief items were analyzed categorically. Family-income adequacy was self-perceived and not standardized across countries. Family history of cervical cancer and vaccination status were self-reported and not verified through clinical records, parents, vaccination cards, schools, or health systems.

Five negatively worded vaccine-perception items retained in the analytic dataset were scored on a seven-point Likert-type response scale from 1 (strongly disagree) to 7 (strongly agree); 4 represented a neutral response. These items were not validated as a unidimensional HPV-vaccine hesitancy scale and were not combined into a total score. Accordingly, Cronbach’s alpha was not used to support a scale interpretation. Item means, standard deviations, and full response distributions were reported descriptively.

### Statistical analysis

Data were analyzed using IBM SPSS Statistics version 25 and independently checked against the deidentified source workbook. Categorical variables were summarized as n/N (%) using item-specific denominators; age and Likert-type items were summarized using means and standard deviations. Obvious typographical variants in categorical responses were harmonized without changing substantive meaning, and spelling variants of refusal reasons were consolidated. Missing or non-applicable responses were not imputed. Imperfect skip logic produced a small number of denominator inconsistencies, which are reported explicitly.

The primary analysis was descriptive. One exploratory complete-case binary logistic regression model examined willingness to accept free HPV vaccination (yes vs. no). The following covariates were entered simultaneously on substantive grounds: age, country, living area, maternal educational status, perceived income adequacy, family history of cervical cancer, HPV awareness, cervical-cancer awareness, screening awareness, HPV-vaccine awareness, and previous HPV vaccination. Egypt, rural residence, school-level maternal education, perceived income inadequacy, absence of family history, and “no” responses were reference categories. Adjusted odds ratios (aORs), 95% confidence intervals (CIs), and two-sided Wald *p* values are reported. Overall model fit was assessed using the likelihood-ratio test. No automated variable selection or multiplicity adjustment was applied; results are hypothesis-generating and should not be interpreted causally or as country-level estimates.

## Results

### Participant characteristics

The sample comprised 300 female middle-school students, with 100 respondents from each country. Mean age was 15.66 years (SD 1.06; range 14–17), and 238/300 participants (79.3%) reported urban residence. Perceived family-income adequacy and family history of cervical cancer were self-reported and require cautious interpretation across settings (Table 2). Country-stratified descriptive results are provided in Supplementary Table S1.

**Table 2:** Participant characteristics.

Characteristic	Category	Value
Country	Egypt	100/300 (33.3%)
	Saudi Arabia	100/300 (33.3%)
	United Arab Emirates	100/300 (33.3%)
Age, years	Mean (SD); range	15.66 (1.06); 14–17
Living area	Urban	238/300 (79.3%)
	Rural	62/300 (20.7%)
Maternal educational status	School graduate	119/300 (39.7%)
	Postgraduate/medical profession	181/300 (60.3%)
Self-perceived family income adequacy	Enough	128/300 (42.7%)
	Not enough	172/300 (57.3%)
Self-reported family history of cervical cancer	Yes	158/300 (52.7%)
	No	142/300 (47.3%)

Values are n/N (%) unless otherwise stated.

### HPV, cervical cancer, screening, and vaccination awareness

Overall, 89/300 participants (29.7%) reported having heard of HPV, 103/300 (34.3%) had heard of cervical cancer, and 49/300 (16.3%) were aware of cervical-screening tests. The HPV information-source item was answered by 90 participants; among them, school nurses were the most frequently reported source (47/90; 52.2%).

HPV-vaccine awareness was reported by 131/298 respondents (44.0%); two responses were missing and were not imputed. Previous HPV vaccination was self-reported by 106/300 participants (35.3%). A total of 197/300 (65.7%) said they would accept HPV vaccination if offered free. Among the 103 who would not accept it, 88 provided a reason; doubt about effectiveness was the most frequent reason after consolidation of spelling variants (50/88; 56.8%) (Table 3). Country-stratified estimates are shown in Supplementary Table S2.

**Table 3:** HPV, cervical cancer, screening, and vaccination-related responses.

Indicator	Result	Missingness or denominator note
Had heard of HPV	89/300 (29.7%)	No missing responses
Answered the HPV information-source item	90/300 (30.0%)	210 did not answer/not applicable
Most frequent source among source-item respondents	School nurse: 47/90 (52.2%)	Denominator is source-item respondents
Reported knowing that HPV can induce disease	90/300 (30.0%)	No missing responses
Had heard of cervical cancer	103/300 (34.3%)	One obvious typographical variant was harmonized to “yes”
Aware of cervical cancer screening tests	49/300 (16.3%)	No missing responses
Aware of the HPV vaccine	131/298 (44.0%)	Two responses missing; not imputed
Self-reported previous HPV vaccination	106/300 (35.3%)	Not verified against records
Reported completing two doses	90/108 (83.3%)	Two more students answered than reported prior vaccination, indicating imperfect skip logic
Would accept HPV vaccination if offered free	197/300 (65.7%)	No missing responses
Would not accept free HPV vaccination	103/300 (34.3%)	No missing responses
Provided a refusal reason	88/103 (85.4%)	15 did not provide a reason
Most frequent refusal reason	Doubt about effectiveness: 50/88 (56.8%)	Spelling variants were consolidated

### Selected HBM-informed beliefs and cues to action

Nearly two-thirds believed that healthy diet or fruit and vegetable intake influences disease risk. Only 11/300 participants (3.7%) reported receiving a recommendation or discussing the topic with a physician, whereas 250/300 (83.3%) reported attending an awareness session (Table 4). These items should be interpreted individually and not as a validated HBM score.

**Table 4:** Selected HBM-informed beliefs and cues to action.

Selected belief or cue-to-action item	Yes, n/N (%)	No, n/N (%)
Believed that a healthy diet influences disease risk	197/300 (65.7%)	103/300 (34.3%)
Believed that a fruit- and vegetable-rich diet influences disease risk	185/300 (61.7%)	115/300 (38.3%)
Had received a recommendation or discussed the topic with a physician	11/300 (3.7%)	289/300 (96.3%)
Reported attending an awareness session	250/300 (83.3%)	50/300 (16.7%)

### Vaccine-related perceptions

Responses to the five negatively worded items were mixed and predominantly neutral or reflected disagreement rather than consistent endorsement of distrust. The highest mean was observed for “People are deceived about vaccine efficacy” (mean 3.97, SD 0.97), which was close to the neutral midpoint of 4. “Vaccine safety data is often fabricated” had a mean of 2.13 (SD 0.56), reflecting overall disagreement (Table 5). Full seven-category distributions are provided in Supplementary Table S3.

**Table 5:** Negatively worded vaccine-perception items, scored from 1 (strongly disagree) to 7 (strongly agree).

Item	Mean	SD
Vaccine safety data is often fabricated	2.13	0.56
Immunizing children is harmful, and this fact is covered up	3.75	0.59
Pharmaceutical companies cover up the dangers of vaccines	3.75	0.76
People are deceived about vaccine efficacy	3.97	0.97
People are deceived about vaccine safety	3.63	1.14

### Exploratory logistic regression

The complete-case model included 298 participants (195 willing and 103 unwilling) and was statistically significant overall [likelihood-ratio *χ*^2^ (12) = 34.99, *p* < 0.001]. Older age, residence in Saudi Arabia or the United Arab Emirates compared with Egypt, reporting that family income was enough, and having heard of cervical cancer were associated with lower adjusted odds of willingness to accept free vaccination (Table 6). Other covariates were not statistically significant. These directions are not interpreted as causal or protective effects because of the cross-sectional design, non-probability recruitment, potential residual confounding, and multiple exploratory coefficients.

**Table 6:** Exploratory complete-case logistic regression for willingness to accept free HPV vaccination (*n* = 298).

Predictor	Comparison/reference	Adjusted OR	95% CI	*p* value
Age	Per 1-year increase	0.72	0.56–0.93	0.012
Country: Saudi Arabia	Egypt	0.37	0.18–0.79	0.010
Country: United Arab Emirates	Egypt	0.36	0.15–0.88	0.024
Urban residence	Rural	0.70	0.31–1.60	0.401
Maternal postgraduate/medical education	School graduate	1.52	0.80–2.92	0.202
Self-perceived income “enough”	Not enough	0.46	0.22–0.93	0.030
Family history of cervical cancer	No family history	1.00	0.55–1.84	0.989
Had heard of HPV	No	1.05	0.59–1.87	0.865
Had heard of cervical cancer	No	0.33	0.19–0.58	<0.001
Aware of cervical screening	No	0.57	0.28–1.16	0.118
Aware of HPV vaccine	No	1.10	0.64–1.89	0.725
Previous HPV vaccination	No	0.89	0.46–1.74	0.736

OR, odds ratio; CI, confidence interval. All covariates were entered simultaneously. The analysis was exploratory; *p* values were not adjusted for multiple comparisons.

## Discussion

This exploratory online survey found limited HPV and cervical-cancer prevention awareness among middle-school girls from Egypt, Saudi Arabia, and the United Arab Emirates. Fewer than one-third had heard of HPV, just over one-third had heard of cervical cancer, and only one in six reported awareness of cervical-screening tests. These sample-specific findings are important as preliminary signals because early adolescence is a key period for developing prevention literacy before routine vaccination opportunities are missed (2–4).

Nearly two-thirds of participants said they would accept HPV vaccination if it were offered free of charge. However, the corrected seven-point response distributions do not support describing distrust as uniformly high. Neutral responses predominated for four negatively worded items, while most participants disagreed that vaccine-safety data are often fabricated. A minority nevertheless endorsed several distrust-related statements, indicating that uncertainty, misinformation, and trust remain relevant communication targets. Affordability and availability may therefore be important, but education should also address safety, efficacy, and source credibility without overstating the level of hesitancy in this sample.

The exploratory logistic model produced several inverse associations, including lower willingness with older age, residence in Saudi Arabia or the United Arab Emirates relative to Egypt, perceived income adequacy, and awareness of cervical cancer. These findings are counterintuitive in places and should be treated as hypothesis-generating rather than explanatory. Awareness may have arisen from prior concern or negative information, perceived income was subjective and not comparable across countries, and the country coefficients may reflect unmeasured differences in recruitment networks, parental influence, or service exposure. The model does not establish that awareness reduces willingness, nor can it rank countries or evaluate policy performance.

The broader descriptive findings converge with regional evidence of limited HPV knowledge and variable vaccine acceptance (5–7, 16, 17). However, that evidence base has largely been built from adult community samples, parents, healthcare-related university students, or single-country studies. In a systematic review of studies from Arab states, very few included adolescents and young adults; most involved clinicians, students in health fields, parents, or adult women (16). More recent studies have added parental and university perspectives (5–7, 17), while a Saudi school-based intervention demonstrated that targeted education can improve secondary-school girls’ knowledge (18). The present study contributes a preliminary multi-country adolescent perspective, but its novelty lies in identifying candidate educational and measurement priorities rather than estimating prevalence or ranking countries.

Among respondents who answered the information-source item, school nurses were the most frequently reported source of HPV information. This finding requires caution because only 90 students answered the item and recruitment may have been connected to school or nursing networks. It nevertheless supports testing the value of school-linked delivery. A feasible intervention could combine an age-appropriate module on HPV, cancer prevention, vaccination, safety, and common misinformation; take-home or digital materials for parents; opportunities to ask trained school nurses or clinicians questions; and referral to locally available vaccination services. Materials should be culturally and linguistically adapted, avoid fear-based messaging, and transparently address uncertainty and adverse-effect concerns. Because parental authorization and service availability shape uptake, adolescent education should be paired with parent engagement and referral pathways rather than delivered as a stand-alone knowledge campaign (4, 18).

Policy and service contexts also matter. Publicly available sources indicate differences in vaccination recommendations and program integration as well as cervical-screening guidance across the three countries (9–11). These policy descriptions provide interpretive context only. They were not measured as participant-level exposures, may not reflect access or implementation during data collection, and cannot be used to explain the observed country coefficients. Future studies should collect school type, city or governorate, parental education, health-insurance or service access, vaccine-policy exposure, and documented vaccination status.

The five negatively worded items do not measure the full construct of HPV-vaccine hesitancy and should not be combined into a scale without validation. Their one-directional wording may increase acquiescence or response-pattern bias among adolescents. Future work should use balanced, multidimensional instruments; conduct cognitive interviews and cross-language adaptation; and test measurement invariance across countries. A staged research program should co-design messages with adolescents, parents, school nurses, and vaccination providers; use stratified school-based sampling that accounts for clustering and country context; and evaluate a co-designed adolescent-plus-parent intervention in a preregistered cluster-randomized trial. Outcomes should extend beyond immediate knowledge to validated vaccine-confidence measures, parent–adolescent decision concordance, and record-verified vaccine initiation and series completion.

This study has several strengths. It focused on adolescents, a group less frequently represented in regional HPV research; used balanced country quotas for preliminary description; reported item-specific denominators; and separated descriptive item reporting from scale-based claims. The revised analysis also reports a single clearly specified exploratory model rather than multiple unplanned comparisons.

Limitations are substantial. Online non-probability snowball sampling likely selected students with internet access, digitally connected families, and parents willing to permit participation. The sample was small per country and mostly urban, so findings cannot be generalized to all middle-school girls, rural adolescents, boys, or out-of-school adolescents. Eligibility, parental consent, age, family history, income adequacy, and vaccination status were self-reported and not independently verified. Several responses were missing, typographical variants required harmonization, and imperfect skip logic produced denominator inconsistencies. The high self-reported family-history and awareness-session frequencies may reflect misunderstanding or selection and require confirmation. The questionnaire was developed for this study and requires further validation; the HBM-informed items did not form full subscales, and the vaccine-perception items should not be interpreted as a validated hesitancy scale. Finally, the cross-sectional design precludes causal inference, and the exploratory regression may reflect residual confounding, selection bias, sparse subgroup patterns, or chance findings from multiple coefficients.

In conclusion, HPV and cervical-cancer prevention awareness appeared limited in this exploratory online sample, although nearly two-thirds expressed willingness to accept free HPV vaccination. Responses to negatively worded vaccine-perception items were mixed and often neutral rather than uniformly distrustful. The study identifies preliminary targets for school-linked, parent-engaged education and a rigorous path for future evaluation. Representative research using validated measures, parent-linked information, and verified vaccination records is needed before effectiveness or policy conclusions are drawn.

## Data Availability

The raw data supporting the conclusions of this article will be made available by the authors, without undue reservation.
