# Supplementary material for: HPV prevention awareness, vaccination willingness, and vaccine-related perceptions among middle-school girls in three Arab countries: an exploratory online survey
Source: Front Public Health. 2026 Aug 3;14:1902409. doi: 10.3389/fpubh.2026.1902409 (PMC13478249; doi:10.3389/fpubh.2026.1902409)
Supplement: Supplementary file 1 [file Table_1.docx]

**Supplementary Material**

**HPV prevention awareness, vaccination willingness, and vaccine-related perceptions among middle-school girls in three Arab countries: an exploratory online survey**

# Supplementary Table S1

Participant characteristics by country. Values are n/N (%) unless otherwise stated.

| **Characteristic** | **Egypt** | **Saudi Arabia** | **United Arab Emirates** | **Overall** |
| --- | --- | --- | --- | --- |
| Age, years | 15.57 (1.10) | 15.58 (1.12) | 15.82 (0.93) | 15.66 (1.06) |
| Urban residence | 84/100 (84.0%) | 64/100 (64.0%) | 90/100 (90.0%) | 238/300 (79.3%) |
| Maternal postgraduate/medical education | 43/100 (43.0%) | 67/100 (67.0%) | 71/100 (71.0%) | 181/300 (60.3%) |
| Self-perceived income “enough” | 67/100 (67.0%) | 59/100 (59.0%) | 2/100 (2.0%) | 128/300 (42.7%) |
| Family history of cervical cancer | 40/100 (40.0%) | 62/100 (62.0%) | 56/100 (56.0%) | 158/300 (52.7%) |

*Note:* Country groups were quota-like non-probability samples and should not be interpreted as nationally representative.

# Supplementary Table S2

Knowledge, awareness, and vaccination-related responses by country.

| **Indicator** | **Egypt** | **Saudi Arabia** | **United Arab Emirates** | **Overall** |
| --- | --- | --- | --- | --- |
| Had heard of HPV | 37/100 (37.0%) | 22/100 (22.0%) | 30/100 (30.0%) | 89/300 (29.7%) |
| Reported knowing HPV can induce disease | 22/100 (22.0%) | 38/100 (38.0%) | 30/100 (30.0%) | 90/300 (30.0%) |
| Had heard of cervical cancer | 42/100 (42.0%) | 34/100 (34.0%) | 27/100 (27.0%) | 103/300 (34.3%) |
| Aware of cervical screening | 26/100 (26.0%) | 12/100 (12.0%) | 11/100 (11.0%) | 49/300 (16.3%) |
| Aware of HPV vaccine | 43/100 (43.0%) | 46/100 (46.0%) | 42/98 (42.9%) | 131/298 (44.0%) |
| Previous HPV vaccination | 12/100 (12.0%) | 34/100 (34.0%) | 60/100 (60.0%) | 106/300 (35.3%) |
| Willing to accept free HPV vaccination | 71/100 (71.0%) | 59/100 (59.0%) | 67/100 (67.0%) | 197/300 (65.7%) |

*Note:* Denominators are item-specific. Country estimates are descriptive and hypothesis-generating only.

# Supplementary Table S3

Full response distributions for negatively worded vaccine-perception items, n/N (%).

| **Item** | **Strongly Disagree** | **Disagree** | **Somewhat Disagree** | **Neutral** | **Somewhat Agree** | **Agree** | **Strongly Agree** |
| --- | --- | --- | --- | --- | --- | --- | --- |
| Vaccine safety data is often fabricated | 0 (0.0%) | 285 (95.0%) | 0 (0.0%) | 7 (2.3%) | 8 (2.7%) | 0 (0.0%) | 0 (0.0%) |
| Immunizing children is harmful, and this fact is covered up | 0 (0.0%) | 8 (2.7%) | 74 (24.7%) | 202 (67.3%) | 16 (5.3%) | 0 (0.0%) | 0 (0.0%) |
| Pharmaceutical companies cover up the dangers of vaccines | 8 (2.7%) | 5 (1.7%) | 70 (23.3%) | 188 (62.7%) | 29 (9.7%) | 0 (0.0%) | 0 (0.0%) |
| People are deceived about vaccine efficacy | 1 (0.3%) | 8 (2.7%) | 73 (24.3%) | 176 (58.7%) | 0 (0.0%) | 42 (14.0%) | 0 (0.0%) |
| People are deceived about vaccine safety | 1 (0.3%) | 83 (27.7%) | 0 (0.0%) | 175 (58.3%) | 24 (8.0%) | 17 (5.7%) | 0 (0.0%) |

*Note:* Items were scored on a seven-point scale from strongly disagree to strongly agree. Each item had N=300 responses. The items were not combined into a total score and should not be interpreted as a validated HPV-vaccine hesitancy scale.
